# Piece-wise linear models of biological systems—Application, analysis, and comparison with nonlinear models using the example of the p53 regulatory module

**DOI:** 10.1371/journal.pone.0243823

**Published:** 2020-12-16

**Authors:** Magdalena Ochab, Krzysztof Puszynski

**Affiliations:** Department of Systems Biology and Engineering, Silesian University of Technology, Gliwice, Poland; Universitat Pompeu Fabra, SPAIN

## Abstract

In this paper, we propose to use a linear system with switching methodology for description and analysis of complex biological systems. We show advantages of the proposed approach over the one usually used, which is based on ODE. We propose the detailed methodology of a full analysis of developed models, including analytical determination of the location and type of equilibrium points, finding an analytical solution, stability and bifurcation analysis. We illustrate the above with the example of the well-known p53 signalling pathway comparing the results with the results of a nonlinear, ODE-based version of the proposed model. The complex methodology proposed by us, especially due to the definition of model structure, which is easy to understand for biologists and medics, may be a bridge for closer cooperation between them and engineers in the future.

## Introduction

In recent years, with growing knowledge of the functioning of organisms, cells and even intracellular signalling pathways it becomes obvious that it will be impossible to fully understand these complex systems, especially their dynamics, without proper mathematical modelling and simulations. Another level of complexity is added by introducing a drug to the system. Each year new drugs are developed and new functions of existed drugs are found [1, 2]. It is impossible to test all possible drug combinations, their scheduling and dosage on real patients, thus so-called “virtual patients” are developed, based on mathematical modelling and model simulations [3]. The idea behind this is to test proposed treatments on these patients first and then test the most promising therapies on real patients.

Over the years two main approaches to mathematical modelling of biological systems (not including spatial aspects) have emerged. Most used are the Ordinary Differential Equations (ODE), as long as a deterministic approach is considered, or those based on the Gillespie algorithm when a stochastic approach is implemented. When the spatial aspect is taken into account we usually use cellular automata or agent-based modelling. The majority of models do not include a spatial aspect, assuming that the population of individuals, cells or intercellular reagents (such as proteins) are well mixed. With the recent development of experimental techniques, such as deep sequencing of single cell [4] or microfluidic cell culture with single protein marking [5], mathematical models of intracellular signalling pathways becomes more and more popular. They allow for a deep insight into cells’ functioning, their response to intra- and extra-cellular stimuli as well as malfunctions responsible for various diseases such as cancer [6].

Each single cell contains hundreds of thousands of molecules: proteins, mRNA and iRNA, each involved in the complicated system of mutual dependencies with thousands of both negative and positive feedback loops. For that reason, we are still far away from a whole-cell model. Usually, only one or two signalling pathways are investigated at the same time thus developed models typically contain at most several or tens of variables. Even then deterministic models contain several or tens of mostly strongly nonlinear ODEs. Analysis of such a system, especially analytical, is complicated or impossible. Even the simplest question of the number and location of the equilibrium points may be unanswered. Thus methods to build simpler to analyze models, which can still reflect complicated nonlinear dynamics, are required. One of the possible solutions may be to use linear models with switching.

Systems with switching are a well-known class of systems, which combine differential equations describing the trajectory evolution over time with step change(s) of structure or parameters of the considered system. In this approach, the system is divided into functional parts with different dynamics. Each subsystem can be used to describe system behaviour in a given condition or time interval, so its model can be relatively simple, even linear. Step change between subsystems allows us to model rapid changes in the system, such as process activation or drug addition in the case of biological systems. A specific type of system with switching is piece-wise linear models, where subsystems are described by a set of linear equations. Such systems can be analyzed not only by numerical simulation but also by analytical methods.

For this reason previous attempts have been made to use a model with switching for the description of a biological system. Snoussi and Thomas in 1993 [7] propose finding two types of stationary points to describe the general behaviour of such systems: regular—to describe behaviour inside a subsystem, for some system state, and singular—to describe system behaviour when the system is changing, e.g., a protein level oscillates. This approach was used in many subsequent studies, where different system modifications and assumptions were made to keep the calculations simple. For example Mestl [8] considers systems where protein level is described only by production and degradation, moreover production is not dependent on system state and degradation is dependent only on the considered protein level. The only switching may be dependent on the system state and only production and degradation parameters may be changed by switching. In a different study [9] the authors were focused on cyclic behaviour and they made an assumption that degradation is steady, independent of system state, which simplifies analysis of cycles’ stability, but is far from real biological system behaviour. Edwards et al. [10] make the further assumption that degradation rate is steady and the same for all the variables, which highly reduces application of their method for real biological systems. There were also attempts to model processes such as transport, protein modification or production based on other variables, such as protein production from mRNA. Among these, Plathe [11] proposed a method involving analysis of dynamics around stationary points by creating a specific matrix. Next the extension of characteristic polynomial for matrix of the differentials must be found, so the calculations are very complex, especially if the system consists of few equations.

In our work we focus on finding a method which allows describing and analysis of real biological systems in which all the reactions such as production, degradation, transport and protein modification may depend on the system state as well as time. Moreover we postulate to use biologically reasonable parameter values, for example different values for the degradation and production rates of different variables. For these reasons some assumptions made in the previous papers do not hold which makes the analysis more complicated. In particular the singular stationary points (SSPs) finding procedures used in the mentioned papers may not indicate the existence of the SSP in the model considered in this paper. Thus the method we propose is described in more detail. In this work we use the well-known p53 signaling pathway which gives us the opportunity to compare our results not only with another ODE-based model but also with well-established biological knowledge.

The implementation of a system with switching for modelling of intracellular processes is in our case supported by the following features of the considered biological system:

- Protein production and degradation often depend on the level of other proteins such as transcription factors. With a low concentration of the transcription factor targeted protein production is slow or does not exist at all. With a high concentration of the transcription factor the production goes at a maximum rate. This may be reflected with high accuracy by switching between two subsystems: one without the production of target protein and the other with maximum rate production. Switching in such a case will depend on the level of the transcription factor. The same idea may be implemented for degradation.
- Many reactions in cells such as complex creation, phosphorylation or ubiquitination are dependent on various enzymes. These reactions follow well known Michaelis-Menten or Hill dynamics. In such case the speed of reaction is slow when the substrate concentration is low and fast with high substrate concentration. This may be easily reflected by switching between two subsystems: one with slow and the second with fast reactions and switching dependent on the substrate level.
- External stimuli such as drug induction to the considered system may be reflected by a time-dependent switch from the subsystem in which there is no drug to the one with the given drug dose.
- One of the main problems with mathematical modelling is fitting the values of parameters. In the case of nonlinear modelling, the problem is exceptionally difficult, due to the need to choose the correct form of nonlinear function and fit the parameters, which sometimes do not have a direct connection with a biological process. If a piece-wise linear system is applied, there are only linear functions, which helps reduce model parameters. Moreover, there are only simple processes like production, degradation or transport. It is easy to define influence of the model parameters on system behaviour, which in nonlinear models is not always so obvious. On the other hand, in systems with switching there is a need to define threshold values, to separate the state of the system into subsystems. However nonlinear functions such as Hill’s function also need the parameter, which stands for the protein concentration (or quantity) at which the function is overloaded. Moreover, a model with different subsystems is easy to relate to biological results, which usually give quantitative results. For example, protein level can be determined by Western Blot presented on gel from electrophoresis with different sized bands, related to the quantity of the protein. Such results are often described quantitatively (low/medium/high level) or by a ratio of control, which can be easily used in the creation of subsystems.

## Materials and methods

### P53 regulatory module—from biology to mathematical model

Protein p53 is known as the *guardian of the genome*, due to its crucial role in cell response to a variety of stress factors, both extracellular and intracellular, especially DNA damage [12]. The main role of protein p53 is an activation of gene expression of proteins responsible for response to DNA damage, in particular proteins responsible for cell cycle blockade, DNA repair, senescence or programmed cell death, called apoptosis. In normal, healthy cells p53 is maintained at a low level by its inhibitor MDM2 and rapidly increases after stress [13]. Abnormalities in the proper functionality of p53 protein, its structure or other proteins involved in its signalling pathway have an influence on proper cell functioning and can result in the emergence of tumour cells.

Due to the significance of the protein p53, there is an abundance of research on its structure, role, interplay with other proteins and abnormal functionality.

**Fig 1:**
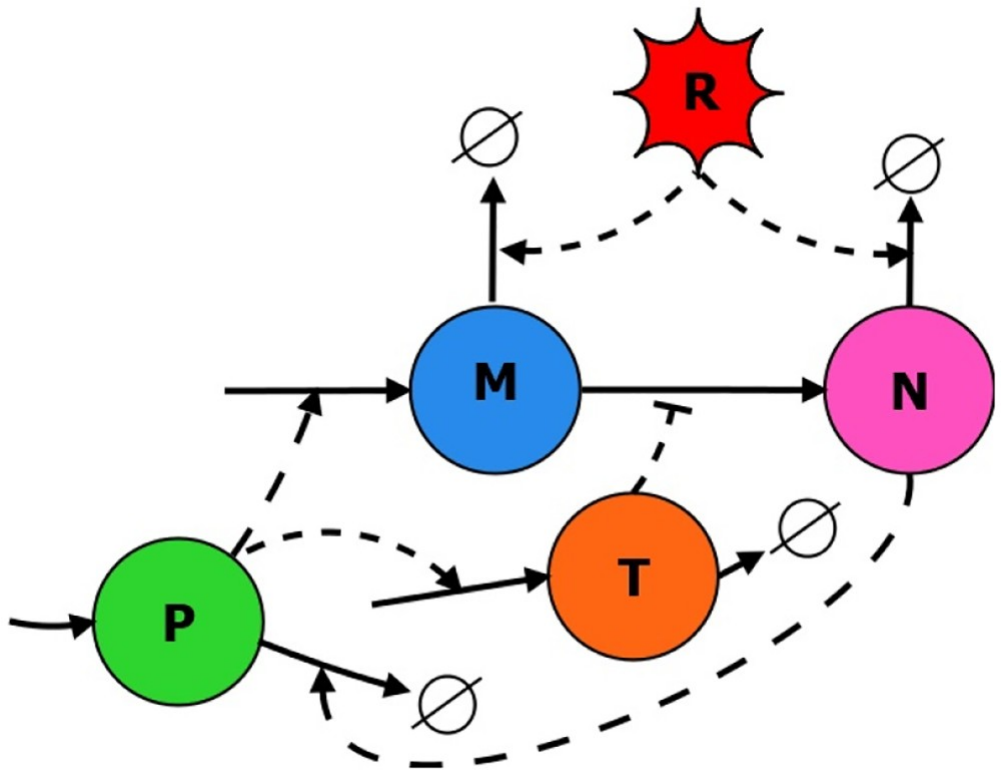
The scheme of the P53 regulatory module model. P stands for nuclear p53, M for cytoplasmic Mdm2, N for nuclear Mdm2 and T for cytoplasmic PTEN.

Mathematical models of p53 dynamics usually focus on the regulatory module consisting of p53 and two proteins transcriptionally dependent on p53: MDM2 and PTEN. Basic dynamics of the p53 regulatory module results from 2 feedback loops: one negative and one positive. Negative feedback includes MDM2 which ubiquitinates p53 and consequently leads to its rapid degradation. Since the p53 protein is located mainly in the nucleus, MDM2 has to be transported to it after translation in the cytoplasm. This may be blocked by the action of the slower, and thus requiring some time for full activation, positive feedback loop which starts from PTEN and goes through PIP3 and Akt [14]. As a result, without DNA damage, we may observe low levels of p53. After DNA damage, we may observe oscillations of the p53 level when only negative feedback is active or further increase of the p53 level to the steady, high level when positive feedback is activated [14]. The interplay between proteins is presented in Fig 1, where *P* denotes p53, *T* denotes PTEN, *M* denotes cytoplasmic MDM2 and *N*—nuclear MDM2.

### Nonlinear model of p53

Over the years many mathematical models of the p53 regulatory module have been proposed [15–17]. Usually, they are very complicated and strongly nonlinear. Presenting the equivalent of such models in the form of a system with switching would lead to a system of multiple linear equations and switchings, which could make it difficult to fully understand the presented approach. Thus we decided to use a simple model consisting of four ODEs, which is the minimal model with full two-feedback dynamics of the p53 regulatory module. The presented model is a simplified version of a full p53 model presented in [18]. The full model contains the equations for gene state, mRNA, proteins, external stimuli by irradiation and two types of siRNA. The simplified version contains only the equations for the main proteins, the gene and mRNA levels were omitted. The influence of the siRNA was neglected. Many intermediate steps and reactions were simplified which results in highly nonlinear terms in the simplified version of the model. We made these simplifications to make the presented analysis clear and easy to follow. All these simplifications were made in such way that the system dynamics, in other words the existence, location and types of equilibrium points as well as cell cycles remains almost the same. The considered model formulas are as follow:

$$\begin{array}{ccc} \frac{dP(t)}{dt} & = & \tilde{p_{1}}-\tilde{d_{1}}\;N{\left(t\right)}^{2}\;P\left(t\right), \end{array}$$ (1)

$$\begin{array}{ccc} \frac{dM(t)}{dt} & = & \tilde{p_{2}}\;\frac{P{\left(t\right)}^{4}}{P{\left(t\right)}^{4}+\tilde{k_{2}^{4}}}-\tilde{d_{2}}\;\left(1+R\right)M\left(t\right)-\tilde{k_{1}}\;\frac{\tilde{k_{3}^{2}}}{\tilde{k_{3}^{2}}+T{\left(t\right)}^{2}}\;M\left(t\right), \end{array}$$ (2)

$$\begin{array}{ccc} \frac{dN(t)}{dt} & = & \tilde{k_{1}}\;\frac{\tilde{k_{3}^{2}}}{\tilde{k_{3}^{2}}+T{\left(t\right)}^{2}}\;M\left(t\right)-\tilde{d_{2}}\;\left(1+R\right)N\left(t\right), \end{array}$$ (3)

$$\begin{array}{ccc} \frac{dT(t)}{dt} & = & \tilde{p_{3}}\;\frac{P{\left(t\right)}^{4}}{P{\left(t\right)}^{4}+\tilde{k_{2}^{4}}}-\tilde{d_{3}}\;T\left(t\right), \end{array}$$ (4)

The subsequent elements of the equations have the same meaning as in the model with switching (Eqs (5)–(8)) and are explained in the next subsection. Parameters of the nonlinear model were marked with a tilde, and their values are in the Table 1, the initial conditions are the same as in the model with switching presented in Table 2.

**Table 1:** Parameters value for the nonlinear model.

Parameter	Value	Parameter	Value
$\tilde{p_{1}}$	8.8 s^−1^	$\tilde{d_{3}}$	3 ⋅ 10^−5^ s^−1^
$\tilde{p_{2}}$	440 s^−1^	$\tilde{k_{1}}$	1.925 ⋅ 10^−4^ s^−1^
$\tilde{p_{3}}$	100 s^−1^	$\tilde{k_{2}}$	1 ⋅ 10^−5^ mol^4^
$\tilde{d_{1}}$	1.375 ⋅ 10^−14^ s^−1^	$\tilde{k_{3}}$	1.5 ⋅ 10^−5^ mol^2^
$\tilde{d_{2}}$	1.375 ⋅ 10^−5^ s^−1^		

**Table 2:** Parameters, thresholds and initial values for the model with switching.

Parameter	Value	Parameter	Value
*p*_1_	8.8 s^−1^	*θ*_*P*_	4.5 ⋅ 10^4^ mol.
*p*_20_	2.4 s^−1^	*θ*_*N*1_	4 ⋅ 10^4^ mol.
*p*_21_	21.6 s^−1^	*θ*_*N*2_	8 ⋅ 10^4^ mol.
*p*_30_	0.5172 s^−1^	*θ*_*T*_	10^5^ mol.
*p*_31_	3.6204 s^−1^	*P*_0_	2.6858 ⋅ 10^4^ mol.
*d*_10_	9.8395 ⋅ 10^−5^ s^−1^	*M*_0_	1.1166 ⋅ 10^4^ mol.
*d*_11_	6.5435 ⋅ 10^−5^ s^−1^	*N*_0_	1.5438 ⋅ 10^4^ mol.
*d*_12_	9.8395 ⋅ 10^−5^ s^−1^	*T*_0_	1.7240 ⋅ 10^5^ mol.
*d*_2_	1.375 ⋅ 10^−5^ s^−1^		
*d*_3_	3 ⋅ 10^−5^ s^−1^		
*k*_10_	1.5 ⋅ 10^−4^ s^−1^		
*k*_11_	1.4713 ⋅ 10^−4^ s^−1^		

### Model with switching

The system with switching corresponding to the nonlinear model presented in Eqs 1–4 consists of four linear equations, which take different parameter values, depending on the subsystems.

Change of the protein p53 level depends on the production (which is steady) and degradation (which rate depends on the nuclear MDM2 (*N*)):

$$\begin{array}{ccc} \frac{dP(t)}{dt} & = & p_{1}-d_{1}^{*}\left(N\left(t\right)\right)\;P\left(t\right). \end{array}$$ (5)

Cytoplasmic MDM2 level changes depend on the production, that is regulated by p53 (*P*), degradation and transport (regulated by PTEN (*T*)). External stress induces degradation of MDM2, which is included in the model by the parameter *R*, which linearly increases the degradation rate.

$$\begin{array}{ccc} \frac{dM(t)}{dt} & = & p_{2}^{*}\left(P\left(t\right)\right)-d_{2}\;\left(1+R\right)\;M\left(t\right)-k_{1}^{*}\left(T\left(t\right)\right)\;M\left(t\right). \end{array}$$ (6)

An equation describing changes of the nuclear MDM2 level is similar to the equation for its cytoplasmic form, however, it does not include production. Increase of nuclear MDM2 results from transport from cytoplasm and decrease comes from degradation, which can be elevated by stress *R*.

$$\begin{array}{ccc} \frac{dN(t)}{dt} & = & k_{1}^{*}\left(T\left(t\right)\right)\;M\left(t\right)-d_{2}\;\left(1+R\right)\;N\left(t\right). \end{array}$$ (7)

Protein PTEN level is dependent on the production rate (dependent on the p53 level) and degradation, which is steady:

$$\begin{array}{ccc} \frac{dT(t)}{dt} & = & p_{3}^{*}\left(P\left(t\right)\right)-d_{3}\;T\left(t\right). \end{array}$$ (8)

The parameters of the model, whose values depend on the system state are denoted by an asterisk (*). To determine their values in specific subsystems, we introduce four thresholds: *θ*_*P*_, *θ*_*N*1_, *θ*_*N*2_ and *θ*_*T*_ and four switching variables, that describe the state of the system concerning these thresholds:

$$\begin{array}{ccc} Z_{P} & = & \left\{ \begin{array}{c} 0\;\text{for}\;P<\theta_{P}\\ 1\;\text{for}\;P\geq \theta_{P}, \end{array}\right. \end{array}$$ (9)

$$\begin{array}{ccc} Z_{N1} & = & \left\{ \begin{array}{c} 0\;\text{for}\;N<\theta_{N1}\\ 1\;\text{for}\;N\geq \theta_{N1}, \end{array}\right. \end{array}$$ (10)

$$\begin{array}{ccc} Z_{N2} & = & \left\{ \begin{array}{c} 0\;\text{for}\;N<\theta_{N2}\\ 1\;\text{for}\;N\geq \theta_{N2}, \end{array}\right. \end{array}$$ (11)

$$\begin{array}{ccc} Z_{T} & = & \left\{ \begin{array}{c} 0\;\text{for}\;T<\theta_{T}\\ 1\;\text{for}\;T\geq \theta_{T}. \end{array}\right. \end{array}$$ (12)

There is no threshold for *M*, because there is no process activated by cytoplasmic MDM2. As a result state space is divided into 12 subsystems, in each we have a different set of parameters values. Values of parameters are a summation of the basic and induced process rate, what can be easily denoted with the use of the switching variables:

$$\begin{array}{ccc} p_{2}^{*} & = & p_{20}+p_{21}\;Z_{P}, \end{array}$$ (13)

$$\begin{array}{ccc} p_{3}^{*} & = & p_{30}+p_{31}\;Z_{P}, \end{array}$$ (14)

$$\begin{array}{ccc} d_{1}^{*} & = & d_{10}+d_{11}\;Z_{N1}+d_{12}\;Z_{N2}, \end{array}$$ (15)

$$\begin{array}{ccc} k_{1}^{*} & = & k_{10}-k_{11}\;Z_{T}, \end{array}$$ (16)

with the assumption that *k*_10_ > *k*_11_. Values of the model parameters are presented in the Table 2.

### Dynamics of the p53 module

**Fig 2:**
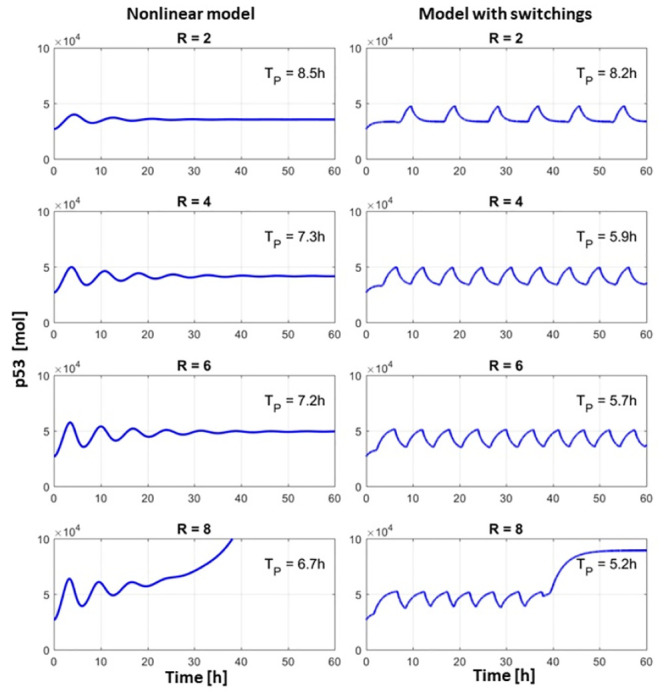
Time courses of nonlinear model and model with switching for different stress levels. *T*_*P*_—period of oscillations.

In a normal, unstressed cell p53 level is low and both models predict such behaviour. After stress, the cell response is activated by the increase of degradation rate of MDM2. As a result of the MDM2 level drop, p53 level increases and processes, such as DNA repair, can be activated. At the same time, the high level of p53 increases the production rate of MDM2 and the negative loop starts to play its role, resulting in p53 level oscillations. P53 oscillations are observed in the biological data [19] and in both models: piece-wise linear and nonlinear (Fig 2). With an increase of stress level, the period of oscillation shortens, which is observed in biological data [20, 21], and in the proposed models (Fig 2). Even a minor increase in the p53 level induces cell cycle blockade and DNA repair, but for apoptosis activation, higher accumulation of p53 is needed.

In the case of higher stress, the p53 oscillation period is smaller, so the accumulation of p53 is high enough to induce positive feedback by activating PTEN expression. PTEN inhibits transport of MDM2 to the nucleus, and subsequently p53 level increases to high values, responsible for apoptosis activation. In biological results p53 in apoptotic cells is 3 times higher than in normal cells [22], which we model in the system with switching (Fig 2, bottom right). However in the nonlinear model, due to its simplification, the saturation is at a much higher level (Fig 2, bottom left). One can notice, that in the case of the nonlinear model oscillations are dampened, which is the results of model simplification. In the full model presented in [18] one can see that the p53 level oscillations are not dampened.

## Results and discussion

**Fig 3:**
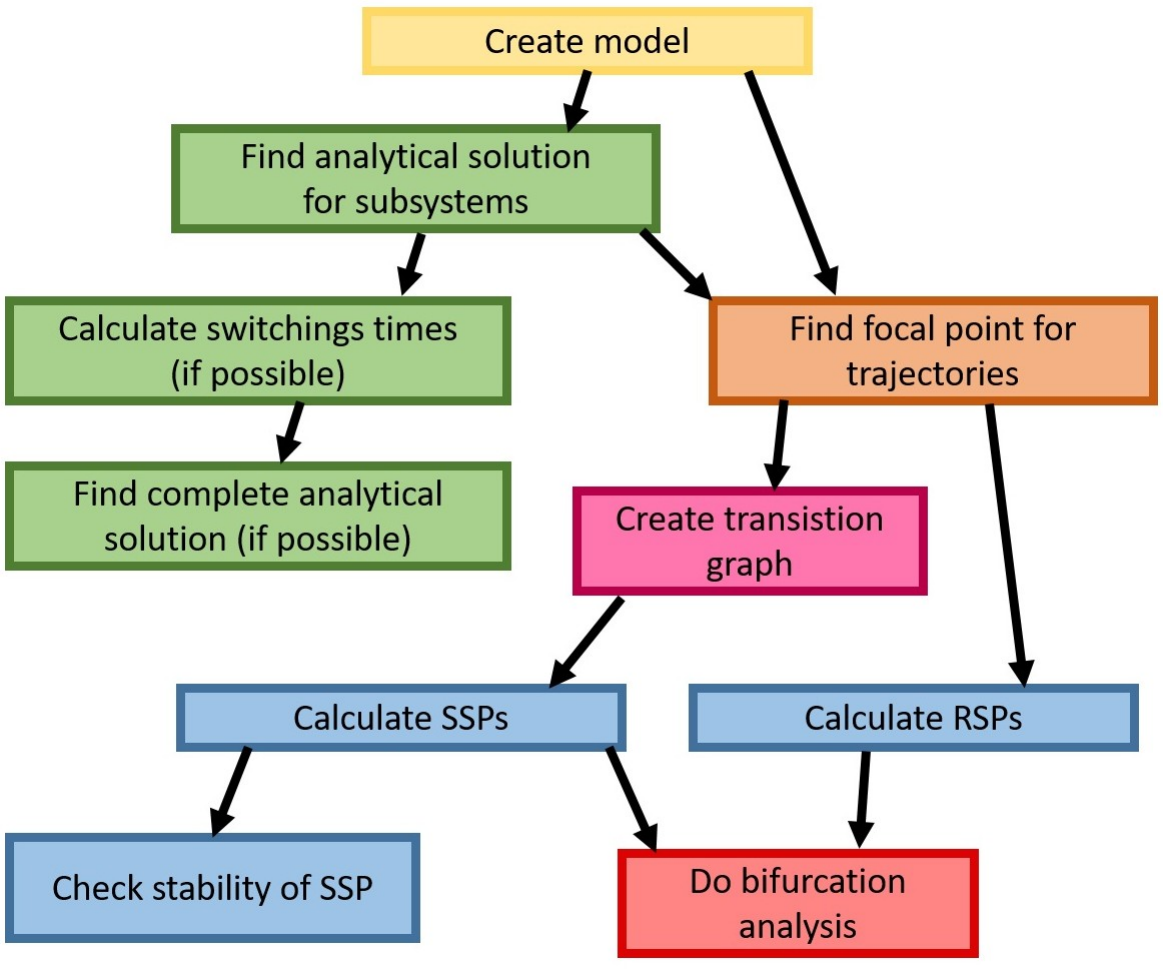
Scheme of the proposed, complex analysis of the systems with switching.

Models with switching are a well-known group of models in automatic control. Due to division into subsystems, even complex dynamics of the system may be described by linear equations inside the particular subsystems. Based on this fact we propose a complex method of analysis of such models (Fig 3).

The proposed method works for piece-wise linear differential equation (PLDE) models, where switching between subsystems depends on the state of systems. The first step of the algorithm is to find an analytical solution for all subsystems. Usually, subsystems differ with respect to the model parameters, but not the structure, so the analytical solution for subsystems have the same formula with different parameter values. In some models, where the analytical formula is not very complicated, is possible to find times of switching with respect to the initial conditions. Having times of switching we can determine complete analytical solution for whole system. Due to linear equations describing system dynamics in each subsystem, we can find focal points for trajectories starting in each subsystem. Focal points can be determined using the analytical solution, by finding the solution for time going to infinity or by solving systems of equations with differentials in ODEs equal to zero. The focal point can be used for creation a transition graph, which presents the qualitative dynamics of the system and supports searching for stationary points and limit cycles. In PLDE models two types of stationary points may exist: regular stationary points (RSPs) are located inside subdomains and singular stationary points (SSPs) are located on the thresholds or crossing of two or more thresholds. The occurrence of RSP results in stabilization of time courses at a given point and occurrence of SSP results in oscillation when trajectories pass through subsystems around the SSP.

The algorithm for determining the localization of RSP and SSP takes into account only a very simple model formulation: just production and degradation [8, 11, 23, 24], so if we want to include processes such as transport or change of one substance into another, which was the case in our model, the methods must be modified. In short, due to the more complicated model, it is not possible to apply Logoid-Jacobian’s method to determine subspaces, that can include a SSP. As a result, all the existed crossing of the thresholds must be analyzed in terms of the existence of a SSP.

The next step of the proposed analysis scheme is the determination of the stability of the calculated SSP. Stability point types can be analogous to those in nonlinear systems without switching (node, saddle, centre). A full description of system behaviour with respect to given parameter values can be achieved by bifurcations analysis and determination of the range of parameters, for which different types of behaviour can be observed. Below we explore the proposed scheme point by point basing on the proposed model of the p53 regulatory module.

### Analytical solutions

The first step of analysis is finding an analytical solution for the model in each subsystem of the PLDE model. In the analyzed case the PLDE model consists of the set of linear equations with the same structure in each subsystem (Eqs 5–8) but different parameters values (Eqs 13–16). We solved the differential equation describing the level of p53 (Eq 5) in the following way: first please notice that in the given subsystem $d_{1}^{*}\left(N\left(t\right)\right)$ is constant so we will denote that by $d_{1}^{*}$. Then introduce

$$\begin{array}{ccc} x(t) & = & P\left(t\right)-\frac{p_{1}}{d_{1}^{*}} \end{array}$$ (17)

so

$$\begin{array}{ccc} \frac{dx(t)}{dt} & = & \frac{dP(t)}{dt}. \end{array}$$ (18)

Calculating P(t) from Eq 17 and substituting it to Eq 5 we receive:

$$\begin{array}{ccc} \frac{dx(t)}{dt} & = & p_{1}-d_{1}^{*}\;\left(x\left(t\right)+\frac{p_{1}}{d_{1}^{*}}\right) \end{array}$$ (19)

which gives

$$\begin{array}{ccc} \frac{dx(t)}{dt} & = & -d_{1}^{*}\;x\left(t\right) \end{array}$$ (20)

which may be solved by the method of separating variables. The equation for PTEN (Eq 8) can be solved the same way. Solving equations for MDM2 (Eqs 6 and 7) is similar but is more difficult due to the necessity of solving an inhomogeneous differential equation. To solve them we used the method of variation of parameters. By using the methods mentioned above we receive the general solution for the p53 regulatory module model for each subsystem as follow:

$$\begin{array}{ccc} P(t) & = & \frac{p_{1}}{d_{1}^{*}}+(P_{0}-\frac{p_{1}}{d_{1}^{*}})e^{-d_{1}^{*}t}, \end{array}$$ (21)

$$\begin{array}{ccc} M(t) & = & \frac{p_{2}^{*}}{d_{2}\left(1+R\right)+k_{1}^{*}}+(M_{0}-\frac{p_{2}^{*}}{d_{2}\left(1+R\right)+k_{1}^{*}})e^{-(d_{2}\left(1+R\right)+k_{1}^{*})t}, \end{array}$$ (22)

$$\begin{array}{ccc} N(t) & = & \frac{k_{1}^{*}p_{2}^{*}}{d_{2}\left(1+R\right)\left(d_{2}\left(1+R\right)+k_{1}^{*}\right)}-(M_{0}-\frac{p_{2}^{*}}{d_{2}\left(1+R\right)+k_{1}^{*}})e^{-(d_{2}\left(1+R\right)+k_{1}^{*})t}\\ & + & (N_{0}+M_{0}-\frac{p_{2}^{*}}{d_{2}\left(1+R\right)})e^{-d_{2}\left(1+R\right)t}, \end{array}$$ (23)

$$\begin{array}{ccc} T(t) & = & \frac{p_{3}^{*}}{d_{3}}+(T_{0}-\frac{p_{3}^{*}}{d_{3}})e^{-d_{3}t}. \end{array}$$ (24)

Please notice that the value of parameters with an asterisk (*) depend on the system state, so their values are different in each domain.

### Focal points

The focal point is the theoretical target point for all trajectories starting from the given subsystem. However, one has to remember that this definition does not take into account the localization of the threshold values. If the focal point is localized outside the given subsystem, trajectories will hit the threshold thus the model parameters and by that, the attraction pool will change. Based on the analytical solution, the focal point for each subsystem can be calculated. For the considered system the general formulas (in each subsystem) for focal point location are as follow:

$$\begin{array}{ccc} & & \lim_{t\to \infty }P\left(t\right)=\frac{p_{1}}{d_{1}^{*}}, \end{array}$$ (25)

$$\begin{array}{ccc} & & \lim_{t\to \infty }M\left(t\right)=\frac{p_{2}^{*}}{d_{2}\left(1+R\right)+k_{1}^{*}}, \end{array}$$ (26)

$$\begin{array}{ccc} & & \lim_{t\to \infty }N\left(t\right)=\frac{k_{1}^{*}p_{2}^{*}}{d_{2}\left(1+R\right)\left(d_{2}\left(1+R\right)+k_{1}^{*}\right)}, \end{array}$$ (27)

$$\begin{array}{ccc} & & \lim_{t\to \infty }T\left(t\right)=\frac{p_{3}^{*}}{d_{3}}. \end{array}$$ (28)

Due to different values of switching parameters (*), localization of focal points is different in each subsystem. If the focal point is localized inside the given subsystem, trajectories inside this subsystem will try to reach it and stay in the regular stationary point. Please notice that this does not have to concern all trajectories. For some initial conditions, time courses on the way to the focal point can hit the threshold and jump to another subsystem.

### Switching times

Because in the considered case the formulas for p53 (Eq 21) and PTEN (Eq 24) are quite simple, we can calculate the time of switch *t*_*Ps*_ in which *P*(*t*) = *θ*_*P*_

$$\begin{array}{c} t_{Ps}=\frac{1}{d_{1}^{*}}ln(\frac{P_{0}\;d_{1}^{*}-p_{1}}{\theta_{P}\;d_{1}^{*}-p_{1}}) \end{array}$$ (29)

and time of switch *t*_*Ts*_ in which *T*(*t*) = *θ*_*T*_

$$\begin{array}{c} t_{Ts}=\frac{1}{d_{3}}ln(\frac{T_{0}\;d_{3}-p_{3}^{*}}{\theta_{T}\;d_{3}-p_{3}^{*}}). \end{array}$$ (30)

Considering various parameter values and initial points one may notice that, for the threshold *θ*_*P*_, if:

- $P_{0}=p_{1}/d_{1}^{*}$—system at *t* = 0 is in the focal point, therefore in steady state,
- $\theta_{P}=p_{1}/d_{1}^{*}$—focal point is localized at the threshold values, thus the example trajectory will reach the threshold, but it will not cross it and there will be no switch,
- $P_{0}>\theta_{P}\wedge p_{1}/d_{1}^{*}<\theta_{P}$ or $P_{0}<\theta_{P}\wedge p_{1}/d_{1}^{*}>\theta_{P}$—trajectory will reach the threshold at the given time (Eq 29),
- $P_{0}>\theta_{P}\wedge p_{1}/d_{1}^{*}>\theta_{P}$ or $P_{0}<\theta_{P}\wedge p_{1}/d_{1}^{*}<\theta_{P}$—trajectory will go to focal point, which is inside the subsystem in which it is already, so it will not reach the threshold *θ*_*P*_.

Similar conditions can be analyzed for the threshold *θ*_*T*_. For thresholds *θ*_*N*1_ and *θ*_*N*2_ determining the general formula for values of switching times is impossible because of the too complicated formula of analytical solution (Eq 23). It may be done only for some specific conditions such as:

$$\begin{array}{c} M_{0}=\frac{p_{2}^{*}}{d_{2}\left(1+R\right)+k_{1}^{*}} \end{array}$$ (31)

or

$$\begin{array}{c} N_{0}+M_{0}=\frac{p_{2}^{*}}{d_{2}\left(1+R\right)} \end{array}$$ (32)

### Transition graphs

**Fig 4:**
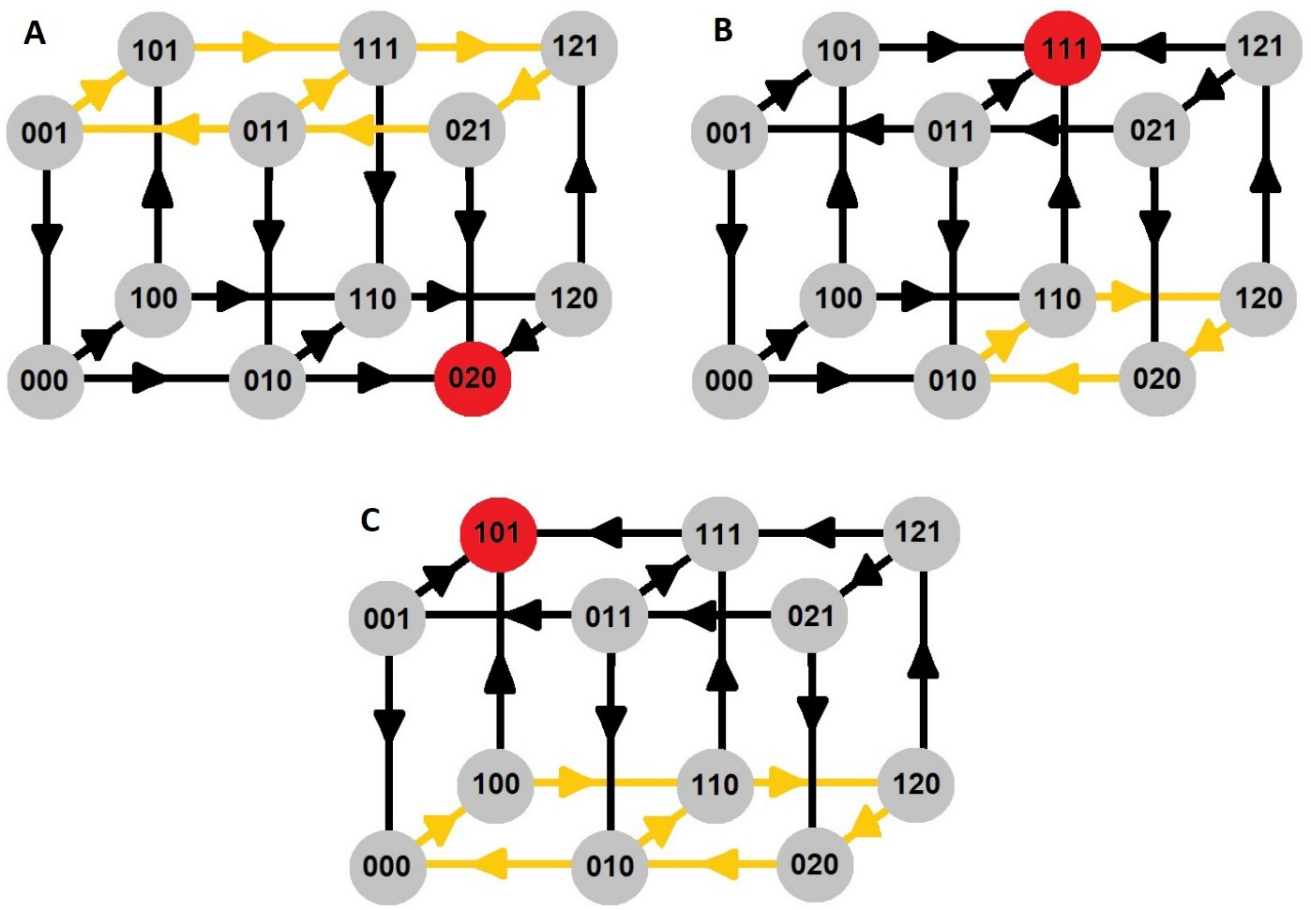
Transition graphs for different stress levels. A: *R* = 0 a.u.; B: *R* = 1.5 a.u.; C: *R* = 9 a.u. Red dot indicates RSP location, yellow arrows indicate closed sequences and by that possible limit cycle location. 0, 1 and 2 indicates if the given variable [P, N, T] is bellow (0) or above (1) its threshold. 2 indicates that N is above the second threshold.

Based on the focal points we can create a transition graph, which presents subsystems and the transitions between them. Simply, for the given subsystem we check in which domain its focal point is located by using Eqs 25–28. Then we join the checked subsystem (source) with the subsystem in which we localized the focal point (destination) by arrow. By repeating this for all subsystems we receive transition graph. Transition graphs are very useful in the description of qualitative behaviour of the system, as we presented previously [25]. In the analyzed p53 model we have 12 subsystems, which we denote using a vector of three elements, describing the level of proteins (*P*, *N* and *T*) in relation to their threshold values: *θ*_*P*_, *θ*_*N*1_, *θ*_*N*2_ and *θ*_*T*_. In the proposed notation 0 means that the given protein level is below threshold and 1 means the protein level is above threshold. The second element of the vector can take values 0, 1 or 2 due to two thresholds for nuclear MDM2. The transition between subsystems will be different in the case of a different external stress level. In a system without any stress, all trajectories go to the subsystem with low p53 and PTEN and high nuclear MDM2, denoted as {020} (Fig 4A). Because we have no arrows leaving this subsystem, so we can conclude, that inside it we have a focal point that is a regular stationary point. For stress of 1.5 a.u. trajectories lead to the subsystem with a medium level of nuclear MDM2, and high level of p53 and PTEN, thus the RSP is localized in {111} (Fig 4B). In the case of high stress—9 a.u. all trajectories lead to the subsystem with high p53 and PTEN and low nuclear MDM2 level. RSP is localized in subsystem {101} (Fig 4C). Additionally, one can notice closed sequences between subsystems on the transition graphs (marked on yellow), this suggests that there is an SSP with a stable limit cycle, however, it has to be confirmed by further analysis.

### Stationary points

Two types of stationary points may exist in the PLDE models: RSPs and SSPs. RSPs are localized inside the considered subsystem and attract trajectories, which stabilize at those points. The system in each domain is described by linear equations so it is quite easy to determine focal point localization. After calculation of the focal point coordinates, one has to check if it is indeed the RSP, by confirming that it is included inside the analyzed subsystem. All RSPs in PLDE models are asymptotically stable. It is important to notice, that the attraction pool for a given RSP does not have to overlap with the whole subsystem. In some cases, trajectories on the way to RSP reach the threshold, where parameters and focal point change.

Calculation of the RSP localization gives information about the protein levels in the steady state. Based on transition graphs, one can notice in which subsystems RSP can exist—the ones from which we have no leaving arrows (Fig 4, marked by red color). In our model, localization of RSPs depends on external stress (*R*). For a chosen stress value, we can calculate focal points in each domain using Eqs 25–28. Please notice that the value of the * parameters depend on the subsystem described by threshold values *θ*_*T*_, *θ*_*N*1_, *θ*_*N*2_ and *θ*_*T*_. So if the focal point is located inside the domain for which we take the values of * parameters it is the regular stationary point, and trajectories inside this subsystem should aim towards it. We found precise localization of RSPs for stress level equal 0, 1.5 and 9 a.u and present them in the Table 3.

**Table 3:** Protein levels in the RSPs.

Stress (*R*)	Subsystem	P_s_	M_s_	N_s_	T_s_
0 a.u.	{020}	33559	14656	159888	17240
1.5 a.u.	{111}	53714	644381	53800	137920
9 a.u.	{101}	89435	170976	3568	137920

We could also reverse the problem and ask what are the limit values of *R* for which the focal point is located in the given domain in which the RSP can exist. One can notice that the *R* is present only in the Eqs 26–27. Because we have thresholds only on *P*, *N* and *T*, not for *M*, we will use Eq 27. It is quadratic equation in respect to *R*. So for example to find the limits of *R* for which the RSP exist in subsystem {020} one has to solve the quadratic inequality:

$$\begin{array}{c} \theta_{N2}<\frac{k_{1}^{*}p_{2}^{*}}{d_{2}\left(1+R\right)\left(d_{2}\left(1+R\right)+k_{1}^{*}\right)} \end{array}$$ (33)

which after a few transformations gives:

$$\begin{array}{c} \theta_{N2}\;d_{2}^{2}\;R^{2}+\theta_{N2}\;\left(2d_{2}^{2}+d_{2}\;k_{1}^{*}\right)\;R+\theta_{N2}\;\left(d_{2}^{2}+d_{2}\;k_{1}^{*}\right)-k_{1}^{*}\;p_{2}^{*}<0 \end{array}$$ (34)

Knowing that in the subsystem {020}, according to the Eqs 9–16 $k_{1}^{*}=k_{10}$ and $p_{2}^{*}=p_{20}$ and also that *R* ≥ 0 we can find the limits for R. This same Eq 27 may be used for other domains in which RSP exists. Of course $k_{1}^{*}$ and $p_{2}^{*}$ has to be taken and inequality created properly to the considered subsystem.

The precise range of the stress values, for which RSP exists in each subsystem are presented below:

- in subsystem {020} RSP exist for *R* ∈ [0, 0.8635) a.u.,
- in subsystem {111} RSP exist for *R* ∈ (1.0322, 1.9154) a.u.,
- in subsystem {101} RSP exist for *R* ∈ (1.9154, + ∞) a.u..

RSP exist in subsystem {020} only for small stress, which does not induce big DNA damage, so the response is not activated. Slightly higher stress values result in an increase of MDM2 degradation, however, its level is still too high to enable high accumulation of p53. As a result, RSP emerges in the subsystem {111}, which is characterized by medium p53 level. Stress higher than 1.9154 a.u. induces degradation of MDM2 fast enough to significantly decrease MDM2 level and thus accumulation of p53. The RSP in subsystem {101} reflect the apoptotic decision taken in the case of significant DNA damage.

For some range of stress level, beside RSP, singular stationary points (SSPs) may also exist, which results in undamped oscillations of p53 level. Existence and location of SSPs may be determined by the procedure proposed by Mestl and coworkers in a series of articles (e.g. [8]). SSP can exist on every threshold or crossing of thresholds, so even for quite simple models, many regions need to be examined for SSP existence. In [8] Mestl et al. proposed a methodology for reducing the number of analyzed regions, however, it can be used for systems with only production and degradation. Our model includes transport, which introduces delay and thus more complicated dynamics, so we have to analyze all possible regions: single thresholds, junctions of two thresholds and points on the crossing of three thresholds. In the considered model, these regions are described by four variables showing protein levels (*P*, *M*, *N*, *T*) compared to the corresponding thresholds. For example, the region described as Δ(*θ*_*P*_, *M*, *N* > *θ*_*N*2_, *T* < *θ*_*T*_) means that the *P* level is at the *θ*_*P*_ threshold, *M* is at any level (we have no threshold for M), *N* level is greater than second threshold *θ*_*N*2_ and *T* below its threshold *θ*_*T*_. The procedure to calculate SSP is as follows:

1. create full model with switching variables
  $$\begin{array}{ccc} \frac{dP(t)}{dt} & = & p_{1}-\left(d_{10}+d_{11}\;Z_{N1}+d_{12}\;Z_{N2}\right)\;P\left(t\right), \end{array}$$ (35)
  $$\begin{array}{ccc} \frac{dM(t)}{dt} & = & p_{20}+p_{21}\;Z_{P}-\left(k_{10}-k_{11}\;Z_{T}\right)\;M\left(t\right)-d_{2}\;\left(1+R\right)\;M\left(t\right), \end{array}$$ (36)
  $$\begin{array}{ccc} \frac{dN(t)}{dt} & = & \left(k_{10}-k_{11}\;Z_{T}\right)\;M\left(t\right)-d_{2}\;\left(1+R\right)\;N\left(t\right), \end{array}$$ (37)
  $$\begin{array}{ccc} \frac{dT(t)}{dt} & = & p_{30}+p_{31}\;Z_{P}-d_{3}\;T\left(t\right). \end{array}$$ (38)
2. for given region, for example Δ(*θ*_*P*_, *M*, *θ*_*N*2_, *T* < *θ*_*T*_) create equation describing stationary state. In this region *P* is equal *θ*_*P*_, *N* is equal *θ*_*N*2_ (so *Z*_*N*1_ = 1) and *T* is smaller then *θ*_*T*_, so *Z*_*T*_ is equal to 0. *Z*_*N*2_ and *Z*_*P*_ are the variables we checking to make sure that SSP is located at the thresholds *θ*_*N*2_ and *θ*_*P*_ respectively. As a result the equations take the following form:
  $$\begin{array}{ccc} 0 & = & p_{1}-\left(d_{10}+d_{11}+d_{12}Z_{N2}\right)\theta_{P}, \end{array}$$ (39)
  $$\begin{array}{ccc} 0 & = & p_{20}+p_{21}Z_{P}-k_{10}M_{s}-d_{2}\left(1+R\right)M_{s}, \end{array}$$ (40)
  $$\begin{array}{ccc} 0 & = & k_{10}M_{s}-d_{2}\left(1+R\right)\theta_{N2}, \end{array}$$ (41)
  $$\begin{array}{ccc} 0 & = & p_{30}+p_{31}Z_{P}-d_{3}T_{s}. \end{array}$$ (42)
3. calculate values of switching variables:
  $$\begin{array}{ccc} Z_{N2} & = & \frac{p_{1}-\left(d_{10}+d_{11}\right)\theta_{P}}{d_{12}\theta_{P}}=0.3224, \end{array}$$ (43)
  $$\begin{array}{ccc} Z_{P} & = & \frac{d_{2}^{2}\theta_{N2}}{k_{10}p_{21}}R^{2}+\frac{2d_{2}^{2}\theta_{N2}+k_{10}d_{2}\theta_{N2}}{k_{10}p_{21}}R\\ & + & \frac{d_{2}^{2}\theta_{N2}+k_{10}d_{2}\theta_{N2}-k_{10}p_{20}}{k_{10}p_{21}} \end{array}$$ (44)
  $$\begin{array}{ccc} & = & 0.0047\cdot R^{2}+0.0603\cdot R-0.0555 \end{array}$$ (45)
  Switching variables have to take values between [0, 1] which is satisfied for *Z*_*P*_ for stress level *R* ∈ (0.8635, 9.9091) a.u.
4. check condition for remaining variables: in this region *T* must be smaller than *θ*_*T*_. This is true for *Z*_*P*_ < 0.6858, so maximum value of *R* must be smaller than 7.7038 a.u.
5. conclude, that SSP may exist in region Δ(*θ*_*P*_, *M*, *θ*_*N*2_, *T* < *θ*_*T*_) for *R* ∈ (0.8635, 7.7038) a.u.

Using the procedure above, we may determine the stress level range in which SSP exist in all 3 possible Δ-regions:

Δ-region (*θ*_*P*_, *M*, *θ*_*N*2_, *θ*_*T*_) contains SSP for *R* ∈ [0; 12.578) a.u.
Δ-region (*θ*_*P*_, *M*, *θ*_*N*2_, *T* < *θ*_*T*_) contains SSP for *R* ∈ (0.8635; 7.7038) a.u.
Δ-region (*θ*_*P*_, *M*, *θ*_*N*2_, *T* > *θ*_*T*_) contains SSP for *R* ∈ [0.7059; 1.0322) a.u..

**Fig 5:**
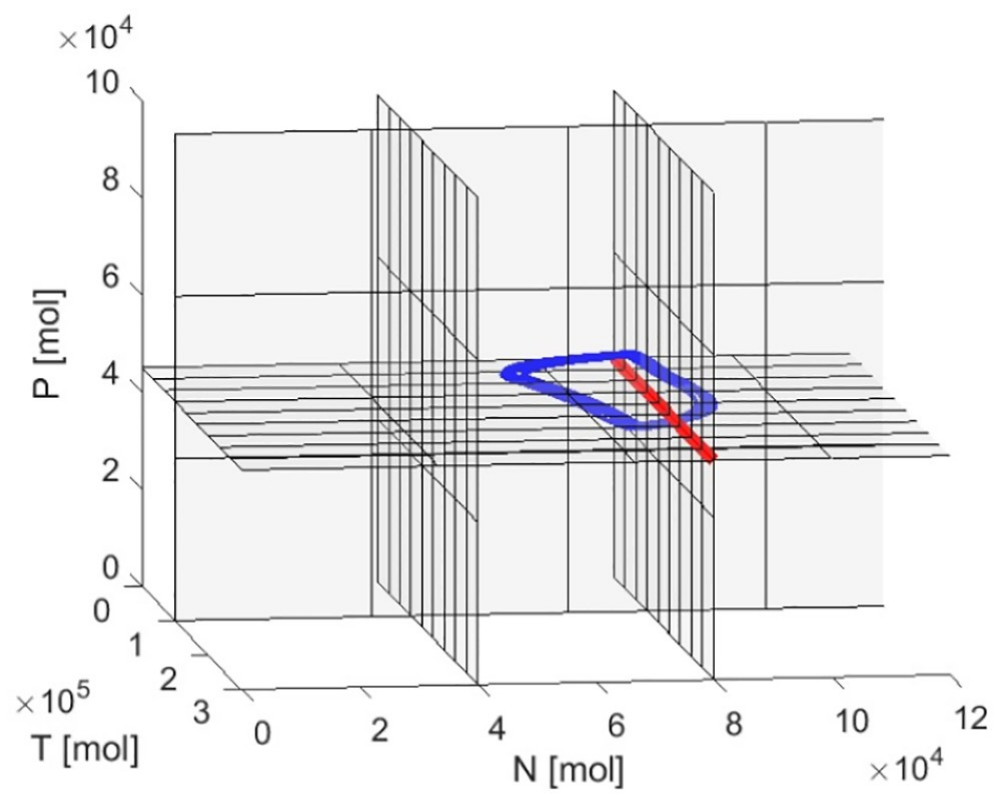
Localization of SSPs in the state space for different stress values. Red line indicates location of the SSPs. Blue line shows exemplary trajectory.

All three SSPs are on the crossing of thresholds *θ*_*P*_ and *θ*_*N*2_ for *T* smaller, equal or higher than threshold *θ*_*T*_. Existence of the SSP causes the oscillations of protein levels. In Fig 5 calculated SSPs are presented in the state space, exactly on the crossing of the thresholds. The blue line shows the example trajectory of the limit cycle for one, chosen stress value and initial condition. In the analyzed system, oscillations result from the negative feedback loop between MDM2 and p53 which has confirmation in the biological results [21].

### Bifurcation diagram

**Fig 6:**
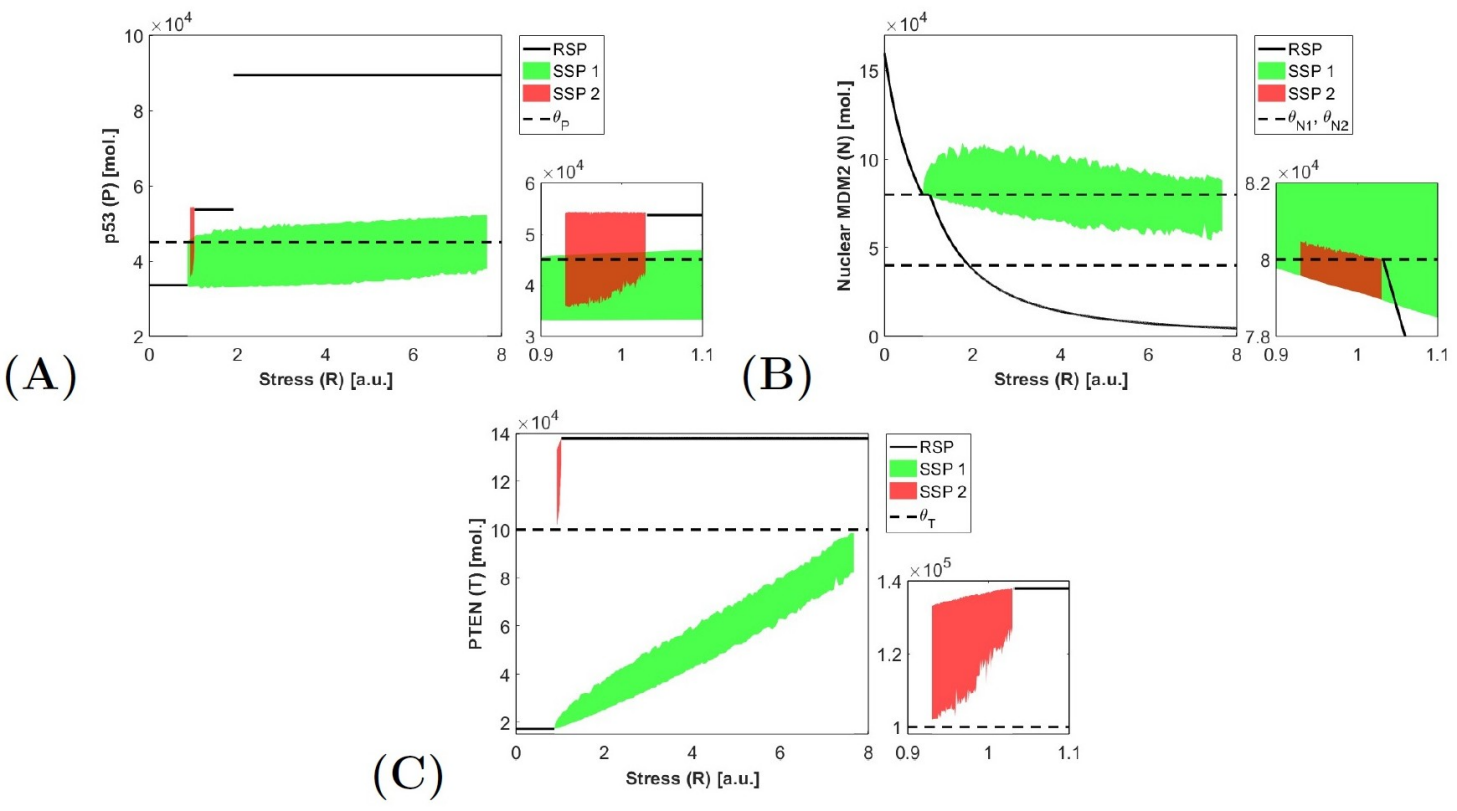
Bifurcation diagrams of protein levels for different stress level. A: Bifurcation diagram for p53 (*P*); B: Bifurcation diagram for MDM2 (*N*); C: Bifurcation diagram for PTEN (*T*). On the right side of the main panels we show the magnification for *R* = (0.9, 1.1)*a*.*u*.

Analysis of the localization of RSPs and SSPs in the considered system indicates that the type of system response and localization of stationary points change with respect to stress value *R*. We have one RSP where the system stabilizes and SSP, around which we have undamped oscillations. As one may notice in the previous paragraph, for some stress levels we have two different stationary points, which suggest that system response depends on the initial conditions and system space is divided into attraction pools. In such cases, a bifurcation analysis may be helpful to better understand the whole system dynamics. We performed it by creating the bifurcation diagrams of protein levels: p53, nuclear MDM2 and PTEN for a wide range of stress values as a bifurcation parameter (Fig 6).

Numerical simulations confirmed oscillation around two SSP: above and below threshold *θ*_*T*_. We do not observe oscillation around SSP located in the crossing of three thresholds *θ*_*P*_, *θ*_*N*2_ and *θ*_*T*_, which suggest that this SSP is not the stable one and the trajectories are attracted by other stationary points.

SSP in region (*θ*_*P*_, *M*, *θ*_*N*2_, *T* < *θ*_*T*_) (denoted as SSP 1—green color) exists for stress value *R* ∈ [0.8635; 7.7038) a.u. which is consistent with analytical results. Numerical results show that protein level oscillates around *θ*_*P*_ and *θ*_*N*2_ and below *θ*_*T*_ (Fig 6).

SSP in region (*θ*_*P*_, *M*, *θ*_*N*2_, *T* > *θ*_*T*_) (denoted as SSP 2 and by red color) is also confirmed by numerical simulation, but only for *R* ∈ [0.95; 1.0322) a.u. This range is slightly smaller than the analytically determined range. This results from the transition of the trajectory in the limit cycle through the threshold *θ*_*T*_ for smaller values of *R*. After reaching the threshold, the trajectory is attracted by the first SSP, so for *R* < 0.95 we have only oscillations below threshold *θ*_*T*_. The range of stress values for which we can observe two SSPs is presented in the magnification on the right side (Fig 6). On the bifurcation diagram for PTEN, one can notice that for *R* smaller than 0.95 oscillation will be reaching threshold *θ*_*T*_.

The above analysis shows that the analytically calculated SSP may be very helpful in the examination of system behaviour. However the existence of SSP does not guarantee that the oscillations of variable levels will be visible in time courses, therefore it has to be checked numerically.

**Fig 7:**
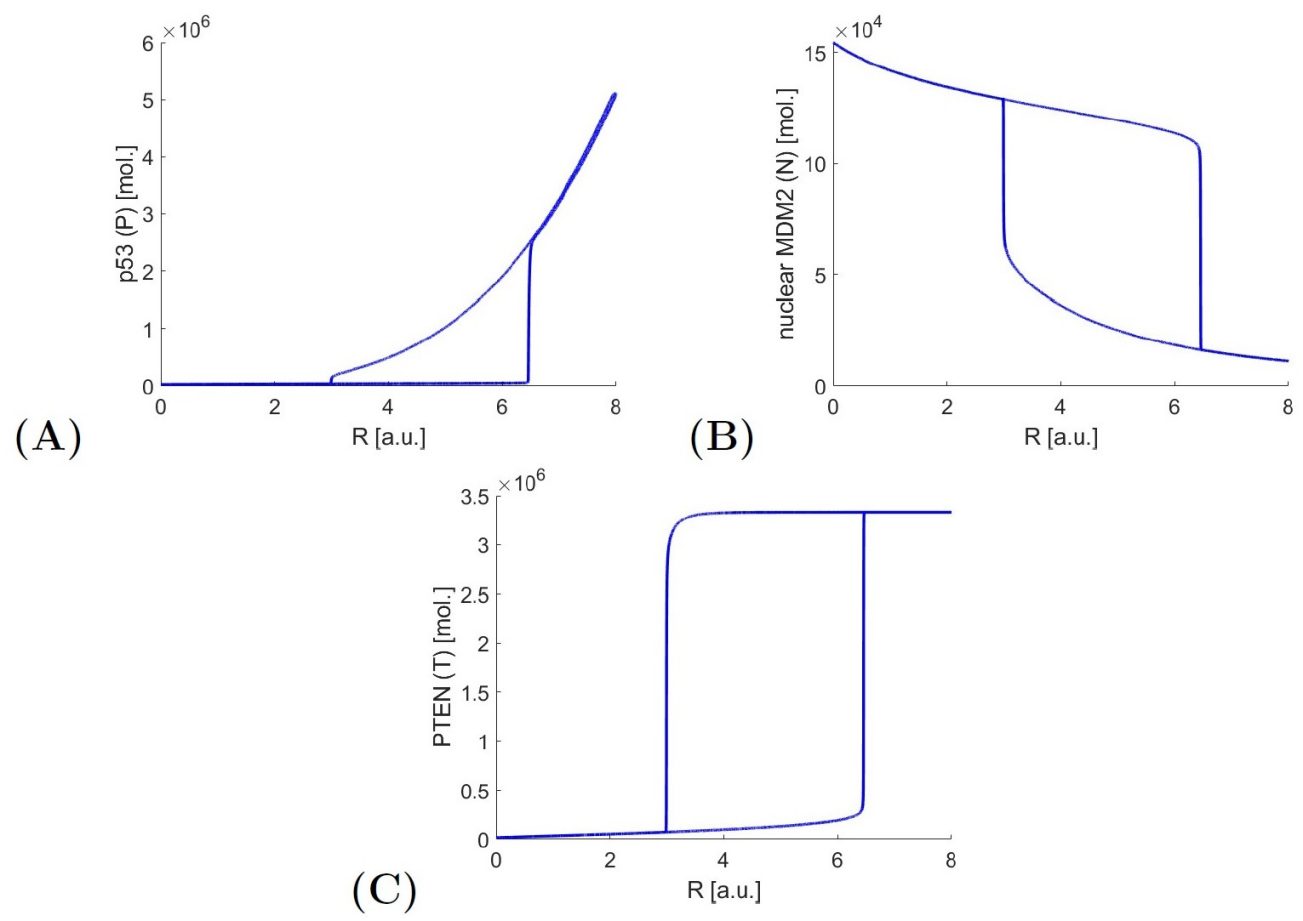
Bifurcation diagrams of protein levels for different stress levels—Nonlinear model. A: Bifurcation diagram for p53 (*P*); B: Bifurcation diagram for nuclear MDM2 (*N*); C: Bifurcation diagram for PTEN (*T*).

Comparing the results of bifurcation analysis of the linear system with switching (Fig 6) to the nonlinear one (Fig 7), one can notice that the oscillation level and the location of apoptotic equilibrium for p53 are much higher in the nonlinear model. As mentioned before this is the result of nonlinear model simplification. Also the range of bifurcation parameter for which we observe system oscillations is different. In the nonlinear system, we can observe the oscillations in a shorter range: from around *R* = 3 to around *R* = 6.5 [a.u.]. In the real, biological cases the wider range is observed thus the system with switching better describes the biologically observed results.

The nonlinear model shows two steady states and one type of oscillations. The system with switching also contains two steady states and the same oscillation type as the nonlinear system (green of Fig 6) but also a second type of oscillations related to the second SSP (red on Fig 6). This second type of oscillations exists only for a small range of stress *R* ∈ [0.7059; 1.0322) a.u. and high PTEN level at this same time. In such conditions, the system oscillates around the threshold for p53 and a second threshold for nuclear MDM2. This situation is very hard to observe in real cells because the high PTEN level causes MDM2 blockade in the cytoplasm thus its nuclear fraction is below even the first threshold and p53 is above its threshold. However, this may suggest that in specifically mutated cells, in which the nuclear entry of the MDM2 is not blocked by PTEN and additionally the p53-MDM2 dependency is weakened, the p53 signalling pathway will still behave in an oscillatory manner instead of reaching apoptotic equilibrium.

## Conclusion

In this work, we proposed to use a linear system with switching methodology to describe and analyze complex biological systems. This allows for a much simpler description of the system, with only linear equations and few threshold definitions, while retaining complex dynamics. The traditional approach based on ODEs requires highly nonlinear equations if we want to keep their number low, or more equations but less complicated to reflect the same level of complexity of dynamics. Nonlinear equations, although sometimes easy to understand for system engineers, are usually hard to follow for biologist and doctors. The simple equations and idea of switching is easier to understand and follow especially taking into account that they operate with the low/medium/high level nomenclature. Because of that, the proposed approach may be helpful in building bridges between the engineers and biologists or medical doctors.

The other advantage of the approach based on systems with switching lies in the need to adjust parameters to biological data. When the minimal approach with highly nonlinear equations is used, we have to find fewer parameters than for a more extended model with more linear equations. The problem is, that instead of simple parameters such as complex creation or dissolution, we have cumulative ones, for which a direct biological explanation is difficult to find. In the extended model we have many parameters, usually too many compared to the amount of biological data to which we fit the model, which makes the results obtained less reliable.

We must consider that the proposed approach results in another fitting problem not present in the ODE approach, which is number and location of thresholds. However, both are usually easy to determine by the analysis of biological data, especially when only qualitative results are required.

The last feature that improves the utility of the proposed approach is that it usually allows for the analytical determination of system properties, not only a numerical solution, as in case of an ODE based approach.

All the properties described above demonstrate the usefulness of the proposed approach.

## Supporting information

S1 File (TXT)
